# Comparing alveolar bone regeneration using Bio-Oss and autogenous bone grafts in humans: a systematic review and meta-analysis

**Published:** 2009-10-10

**Authors:** Alireza Akbarzadeh Baghban, Azam Dehghani, Farzin Ghanavati, Farid Zayeri, Farzam Ghanavati

**Affiliations:** 1 *Department of Biostatistics, Paramedical School, Iranian Center for Endodontic Research, Shahid Beheshti University of Medical Sciences, Tehran, Iran*; 2 *Paramedical School, International Branch of Shahid Beheshti University of Medical Sciences, Tehran, Iran*; 3 *Department of Periodontics, Perio-implant Dept., Dental School, Shahid Beheshti University of Medical Sciences, Tehran, Iran*; 4 *Department of Biostatistics, Paramedical School, Shahid Beheshti University of Medical Science, Tehran, Iran*; 5 *Department of Maxillofacial Surgery, Newcastle, UK*

**Keywords:** Autogenous grafts, Bio-Oss, Bone regeneration, Intrabony defect, Meta-analysis, Systematic review

## Abstract

**INTRODUCTION:** Bone regeneration grafts (BRG) are widely used in the treatment of osseous defects and oral surgery. The various techniques and associated success rates of bone augmentation require evaluation by systematic review and meta-analysis of eligible studies. The aim of this systematic review was to compare alveolar bone regeneration in humans using Bio-Oss and autogenous bone graft.

**MATERIALS AND METHODS:** The computerized bibliographical databases including Pubmed, Google, ScienceDirect and Cochrane were searched for randomized and cohort studies in which autogenous grafts were compared to Bio-Oss in the treatment of periodontal defects. The inclusion criteria were human studies in English that were published 1998-2009. Exclusion criteria included non randomized observation and cohort studies, papers which provided summary statistics without the variance estimates, and studies that did not use BRG intervention alone, were excluded. The screening of eligible studies, assessment of the methodological quality of the trials and data extraction were collected by two observers independently*.* For comparing autogenous grafts used alone against Bio-Oss used alone 5 situations were investigated. Thirteen studies were included in the review which compared autogenous against Bio-Oss, autogenous combined with guided tissue regeneration (GTR) against GTR, Bio-Oss combined with GTR versus GTR, autogenous alone versus Open Flap Debridement (OFD), Bio-Oss versus OFD. In meta-analysis, changes in bone level (bone fill) was used as the measure. Data were analyzed using Bayesian meta-analysis by WinBUGS and Boa software.

**RESULTS:** Only one comparison demonstrated that the difference in bone augmentation between Bio-Oss and OFD was statistically significant.

**CONCLUSION:** There is insufficient evidence to show that Bio-Oss is superior to autogenous grafts in bone augmentation techniques however autogenous bone involves donor site surgery and thus donor site morbidity, so we can conclude that Bio-Oss is better than autogenous for alveolar regeneration. [Iranian Endodontic Journal 2009;4(4):125-30]

## INTRODUCTION

Absent permanent teeth in the adult dentition may be due to dental disease, trauma, iatrogenic mishaps or congenital absence. In addition, they may be lost during maxillofacial surgery due to pathologic lesions such as cancer. The surrounding alveolar bone of the missing tooth is usually minimal; this lack of supporting bone may be due to atrophy, trauma, a failure to develop or surgical resection. Similar phenomenon may be observed through osseous defects which are related to periradicular lesions. In case of tooth missing, dental implants can only be placed if there is sufficient bone to stabilize them adequately; and therefore bone augmentation may greatly assist implant treatment which would otherwise not be a treatment option (1).

The ultimate goal of periodontal therapy is to regenerate periodontal tissues caused by periodontitis (2). For this purpose bone grafts, guided tissue regeneration (GTR) or their combinations have been used (3).

Bone regeneration grafts (BRG) are of most widely used therapeutic strategies for the correction of osseous defects related to periodontal or periradicular lesions. A wide range of graft materials have been applied, including autografts, allografts, xenografts, and synthetic materials (4). This biotechnology can be used for implant placement.

Autografts are taken from an adjacent or remote site in the patient and are used to build up the deficient area and considered to be the gold standard (5).

Xenografts are derived from vital tissues of cow or coral. Bio-Oss is a bovine bone that is processed for the purpose of complete removal of the organic components (6).

There were several observational, controlled, systematic review and meta-analysis studies which showed improvements in clinical parameters such as bone fill of graft materials (7-11). However the regenerative outcomes of osseous tissues remain somewhat inconsistent and are likely to be dependent on multiple factors (1). This systematic review was aimed to compare alveoli bone regeneration using Bio-Oss and autogenous bone graft in humans.

## MATERIALS AND METHODS

***Search Strategy***

A literature search was performed using the Pubmed (Medline), Google, ScienceDirect database and the Cochrane Oral Health Group, this included articles published up to and including March 2009. Hand searching was carried out to find any more related articles. A combination of keywords and MeSH terms were used in our search strategy in an attempt to identify all relevant studies. The search strategy included the following keywords: "intrabony defect" OR "intra bony defect" OR "intra-bony defect"; "bovine xenograft". Following MeSH terms were used in various combinations with the keywords: "periodontal regeneration" OR "periodontal-regeneration"; "infra-bony defect" OR "infrabony defect" OR "infra bony defect" "intra osseous" OR "intraosseous" OR "intra-osseous"; OR "xenograft" OR "Bio-Oss" OR "bovine derived xenograft" OR "bovine derived xenograft". Experts involved in this field of research were contacted to clarify ambiguous information.

***Inclusion criteria:*** All searches were limited to randomize and cohort human studies in English publications that were published in 1998-2009. In addition, this review considered randomized control trials and cohort studies as well as critical reviews of literature examining BRG in the treatment of periodontal osseous defects.

***Exclusion criteria:*** Non-randomized observational studies (*e.g.*, case reports, case series), publications providing summary statistics without variance estimates or data to permit computation, and studies without BRG were excluded.

***Types of participants:*** Patients with missing teeth who may require alveolar bone augmentation prior to or during dental implant placement procedures.

***Types of Intervention***

1. Autogenous *vs.* Bio-Oss.

2. GTR + Autogenous *vs.* GTR.

3. GTR + Bio-Oss *vs.* GTR.

4. Autogenous *vs.* Open Flap Debridement (OFD).

5. Bio-Oss *vs.* OFD.

***Types of outcome measures:*** Despite the variety of reported measurements, only change in bone level (bone fill) was used.

***Screening Methods and Data Extraction:*** All eligible studies were examined and identified for retrieval. Screening of titles and abstracts were completed independently by two authors, based on the following questions:

1. Was the study a randomized control trial?

2. Was the study conducted on patients presenting periodontal intrabony defects?

3. Was the study investigating the efficacy of autogenous and Bio-Oss?

**Figure 1:**
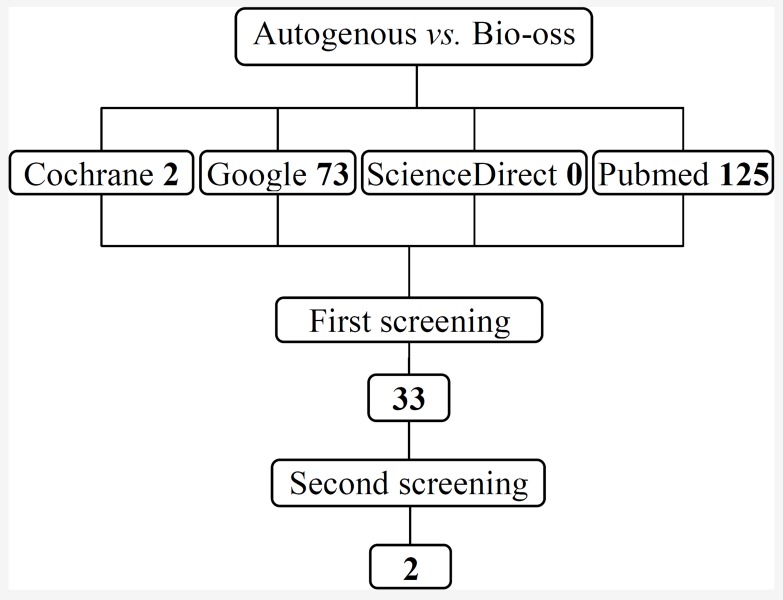
Flow chart of the studies assessed and excluded throughout the various stages of the review.

The full text articles were retrieved when a "yes" or "uncertain" response was given to all screening questions. For studies appearing to meet the inclusion criteria, or those which had insufficient data in the title and abstract to make a clear decision, the full report was obtained. The full reports were assessed independently by two review authors to establish whether the studies met the inclusion criteria. All studies meeting the inclusion criteria then underwent validity assessment and data extraction. The studies which did not meet the inclusion criteria were eliminated.

***Data extraction and Analysis:*** Data were extracted independently by two review authors. Disagreements were resolved by discussion. Where resolution was not possible, a third review author was consulted.

All authors of similar studies were contacted for clarification or missing information. Data were excluded until further clarification was available if agreement could not be reached*. *For each trial the following data were recorded:

 1) year of publication, country of origin and source of study funding;

2) details of the participants including demographic characteristics, source of recruitment and criteria for inclusion;

3) details of the type of intervention;

4) details of the outcomes reported, including method of assessment, and time intervals.

The synthesis of data for outcome measures was based on the experimental design. Mean and variance of estimated alveolar bone regeneration for outcome measures were obtained directly from summary statistics or calculated from the data table. Effect size for each study was calculated as the absolute mean difference between two groups. Heterogeneity was examined by using Geweke's convergence test. A lack of heterogeneity was accepted only when tests yielded insignificant statistics. Few papers were found adequate, so data were analyzed using Bayesian meta-analysis methods and WinBUGS software.

## RESULTS

***Study Characteristic:***

1- Autogenous used alone versus Bio-Oss alone: the computerized search strategies located 1299 citations, of which 200 were screened for potentially meeting inclusion criteria, 167 were excluded during the selection process. In the 33 remaining articles, 31 publications provided summary statistics with-out variance estimates or nonrandomized and cohort studies, so only two were accepted (Figure 1).

**Figure 2:**
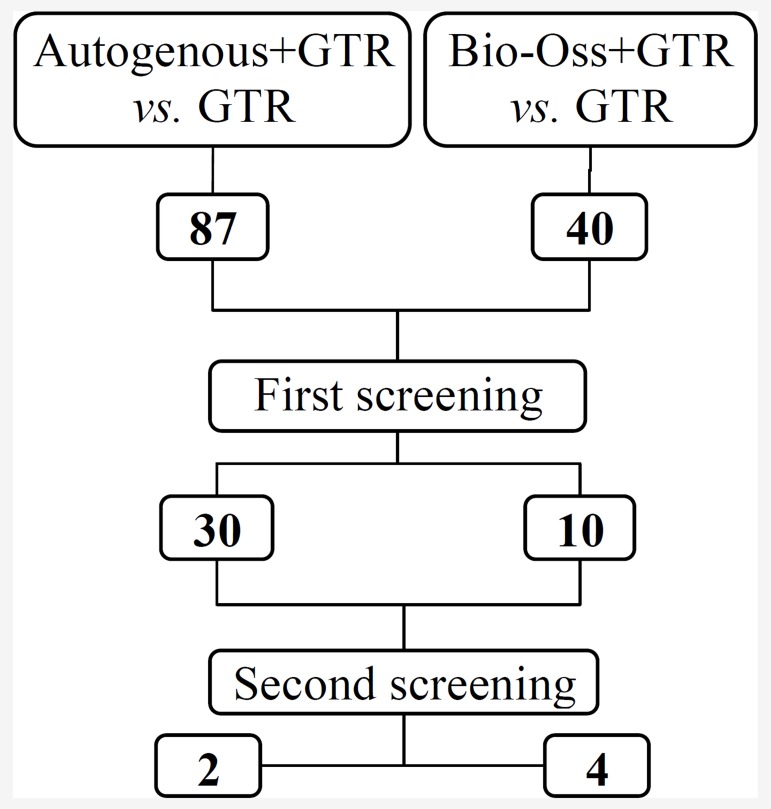
Flowchart of the studies assessed and excluded throughout the review.

2- Autogenous in combination with GTR versus GTR alone: an electronic search found 87 relevant studies, of which 57 were excluded for title and abstracts. After further screening, only 2 studies were included in this systematic review (Figure 2).

3- Bio-Oss combined with GTR versus GTR alone: a total of forty studies evaluated bone augmentation with GTR and Bio-Oss versus GTR alone. Of these, 30 studies were excluded after the first and 6 studies after the second screening (Figure 2).

**Figure 3:**
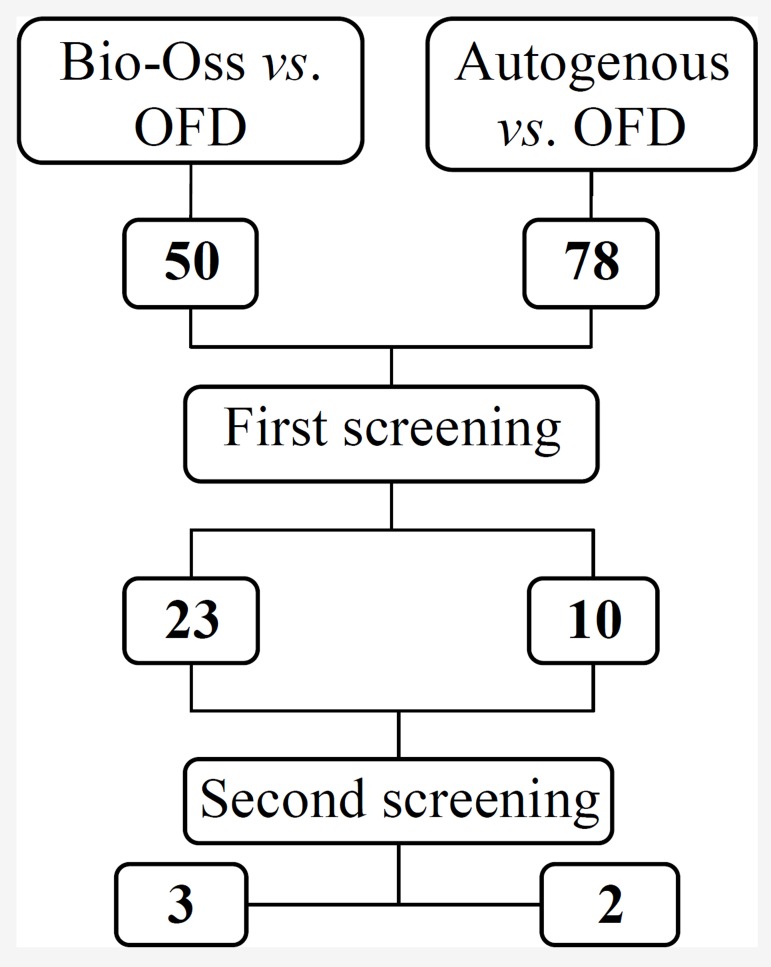
Flowchart of the studies assessed and excluded throughout the review.

4- Autogenous alone versus OFD: ten studies were screened independently from the 78 studies initially found. Two studies compared autogenous with OFD (Figure 3).

5- Bio-Oss used alone versus OFD: search yielded 50 potentially relevant articles. Of these, 27 were excluded in first screening process. In the remaining 23 articles, 20 studies provided summary statistics without variance estimates so three were accepted (Figure 3).

***Treatment of intrabony defects with Autogenous alone versus Bio-Oss alone***

Our search yielded two relevant studies (12,13) (Table 1). The effect size for each study was calculated and examined. A lack of heterogeneity was observed (P>0.05). Data were analyzed; the difference between groups was statistically insignificant (95% CI: -3.678-3.467). Negative treatment difference parameter was found. This means greater increase in bone augmentation with Bio-Oss than autogenous (about 0.04 mm).

***Treatment of intrabony defects with Autogenous and GTR vs. GTR***

Out of the 30 studies, two studies compared the efficacy of autogenous and GTR against GTR (14,15) (Table 1).

Values of 14 and 13.9 mm were found for effect size. Difference between groups and heterogeneity test was statistically insignificant (95% CI: -4.689-7.135). Positive treatment difference parameters were found, it means autogenous and GTR increase bone content compared with GTR alone (1.391 mm).

***Treatment of intrabony defects with Bio-Oss and GTR vs. GTR***

A total of forty studies evaluated the bone augmentation with GTR and Bio-Oss versus GTR alone. Four studies were used for analyzing data (16-19) (Table 1).

Heterogeneity test was statistical insignificant. Examination reported no significant difference between Bio-Oss and GTR against GTR (95% CI: -0.5871-1.522). Bayesian output showed positive treatment difference parameter, it means that, GTR and Bio-Oss increase bone augmentation about 0.4211 mm relative to GTR.

***Treatment of intrabony defects Autogenous vs. OFD***

Ten studies were screened independently. Two studies used Bio-Oss and OFD as two groups for comparing (20,21) (Table 1). A lack of heterogeneity was found (P>0.05). Difference between groups was statistical nonsignificant (95% CI: -0.9553-1.358). Positive treatment difference parameter was found. So, autogenous increase bone about 0.23 mm relative to OFD.

***Treatment of intrabony defects Bio-Oss vs. OFD***

Our search yielded three relevant studies from fifty studies that compared Bio-Oss *vs.* OFD (22-24) (Table 1). An insignificant heterogeneity test result was found (P>0.05). Data were analyzed, the difference between groups was statistically significant (95% CI: 1.115-2.562). Treatment difference parameter was positive. Therefore, Bio-Oss had more bone formation (approximately 1.88 mm) relative to OFD.

## DISCUSSION

The first objective of this systematic review was to compare bone regeneration using Bio-Oss and autogenous bone grafts. However, only two studies were found, so we decided to divide the studies into five broad groups. One similar study (historical trial) tested the possibility of new bone regeneration around implants according to guided bone regeneration (GBR). It showed that bone augmentation was necessary and beneficial for the patient (25). 

**Table 1:** Included studies: Treatment of intrabony defects with Autogenous versus Bio-Oss [12-13], Autogenous+GTR versus GTR [14-15], Bio-Oss+GTR versus GTR [16-19], Autogenous versus OFD [20-21], and Bio-Oss versus OFD [22-24]

Reference Number	Author (Date)	Sample	Size	Means (SE)
**[12]**	Meinjnderi (2005)	Autogenous	5	42.3 (11.5)
		Bio-Oss	5	41.9 (13.1)
**[13]**	Piattelli (2002)	Autogenous	9	29.8 (4.4)
		Bio-Oss	9	29.7 (2.4)
**[14]**	Chen (vertical) (2005)	Autogenous+GTR	13	83.1 (23.8)
		GTR^*^	11	69.1 (27.5)
**[15]**	Chen (horizontal) (2005)	Autogenous+GTR	13	89.7 (19.8)
		GTR	11	75.8 (34.2)
**[16]**	Cornelini (2004)	Bio-Oss+GTR	10	2.1 (1.29)
		GTR	10	0.9 (1.2)
**[17]**	Batista (1999)	Bio-Oss+GTR	11	3.2 (1.4)
		GTR	11	2.9 (1.5)
**[18]**	Paolantonio (2002)	Bio-Oss+GTR	17	5.1 (1.6)
		GTR	17	4 (1.3)
**[19]**	Stavropoulos (2004)	Bio-Oss+GTR	15	2.5 (1)
		GTR	15	2.9 (0.7)
**[20]**	Carroro (1976)	Autogenous	10	0.88 (0.92)
		OFD^**^	10	0.6 (0.89)
**[21]**	Movin (1982)	Autogenous	6	0.86 (0.88)
		OFD	6	0.58 (0.85)
**[22]**	Camargo (2000)	Bio-Oss	11	3.31 (1.1)
		OFD	11	1.8 (1)
**[23]**	Sculean (2001)	Bio-Oss	14	4 (1.3)
		OFD	14	2.1 (1.7)
**[24]**	Stephen (2006)	Bio-Oss	16	4.1 (0.9)
		OFD	16	1.9 (1.1)

* Guided Tissue Regeneration ** *Open Flap Debridement*

Another study evaluated the need for autogenous bone grafting during implant placement in the fresh extraction sockets of maxillary incisors and premolars. Significant differences were reported (26).

A well designed study investigated several implant placement techniques in edentulous patients including iliac crest bone grafting, short implants, and trance-mandibular implants. It showed bone graft technique was better than short and also trans-mandibular implants (27).

Five situations were investigated in this search. The statistical analysis only showed that the difference in bone augmentation between Bio-Oss and OFD was statistically significant. However, the other four comparisons did not demonstrated significant difference. There is good evidence to suggest that Bio-Oss has significant improvement over OFD combined with autogenous when compared to autogenous against OFD and Bio-Oss against OFD. There is insufficient evidence that autogenous is better than Bio-Oss; this necessitates further research. Also there is not enough evidence that autogenous combined with GTR is better than Bio-Oss combined with GTR.

## CONCLUSION

The difference in bone augmentation between Bio-Oss and autogenous was not significant. However, Bio-Oss may be considered superior to autogenous, because autogenous sometimes involves donor site surgery, complications and donor site morbidity.

Once further information becomes available, a meta-analysis can be carried out. 
